# Possession or position games: What is the key in soccer?

**DOI:** 10.5114/biolsport.2024.136086

**Published:** 2024-04-08

**Authors:** Jose A. Asian-Clemente, Borja Muñoz, Jose Vicente Beltran-Garrido, Bernardo Requena

**Affiliations:** 1 Department of Sport sciences, Universidad Pablo de Olavide, Seville, Spain; 2 Football Science Institute, Granada, Spain; 3 Department of Education Science, School of Humanities and Communication Sciences, Universidad Cardenal Herrera-CEU, CEU Universities. Castellon de la Plana, Spain

**Keywords:** Small-sided games, Time-motion, Football, Area per player

## Abstract

The aims of this study were to compare the running performance of possession and position games and to describe the external load of the same position game played on pitches of different dimensions. Using a GPS system (WIMU Pro, RealTrack Systems, Almería, Spain), the running demands of 25 professional soccer players were monitored during 18 possession (without a standardized role position) and 18 positional (with a specific role position) games of 9 vs. 9 + 2 floaters. Each format was developed in small (< 60 m^2^ per payer), medium (60–90 m^2^ per player) and large (> 90 m^2^ per player) sizes. Position games obtained significantly lower distance covered, peak speed and player load values than possession games (all *p* < 0.05). However, position games obtained significantly higher values of maximal acceleration, maximal deceleration, accelerations higher than 3 m · s^−2^ and decelerations lower than -3 m · s^−2^ than possession games (all *p* < 0.05). Likewise, large position games obtained significantly higher values of distance covered, distance covered > 21 km · h^−1^, peak speed and player load than small and medium sizes. Large size also showed significantly higher values of maximal acceleration and deceleration than small size, significantly fewer accelerations and decelerations, and fewer accelerations lower than 3 m · s^−2^ and decelerations higher than -3 m · s^−2^ compared to medium and small size (all *p* < 0.05). Practitioners should keep in mind the use of these games and their size to modify the external load of the players during their training.

## INTRODUCTION

Soccer is the sport with the largest number of participants worldwide, and it has become in the most studied sport in the world [1]. Research has been developed to analyse different factors that affect soccer performance: technical, tactical, mental and physical/physiological areas. Of them all, physical and physiological aspects have received the most attention [2–4]. Previous literature has reported that soccer players run, on average, 9–13 km, with 8–9% covered at speeds above 20 km · h^−1^, and 2–3% at speeds above 25 km · h^−1^ [5]. Likewise, it has been established that during the game, soccer players face significant demands in terms of accelerations and decelerations, contributing to 7–10% and 5–7% of the total player load, respectively [6]. Currently, it is well established that players’ requirements are influenced by their positional roles [5, 7, 8]. For example, a recent study on the last FIFA World Cup Qatar 2022 demonstrated that defensive and central midfielders covered more total distance than players in other positions, while wide midfielders and wide forwards achieved greater distances covered at higher intensities [8]. Similarly, previous studies [6, 9] have shown that acceleration and deceleration demands also vary according to player positions, highlighting that wide midfielders and fullbacks cover more distance while accelerating and decelerating, achieving a greater amount of highintensity acceleration and deceleration. Meanwhile, midfielders perform a greater number of low-intensity accelerations and decelerations. For this reason, some authors consider it necessary to contextualize the running demands of soccer players to avoid underestimating and overestimating overall physical performance metrics [7].

Physical and physiological improvements are achieved when there is greater similarity between soccer training and actual competition [10]. It follows that the best way to achieve these improvements is to incorporate game-related drills and exercises into soccer training sessions. By simulating the intensity, speed, and decision-making aspects of a real game, players can develop the specific skills and fitness required for match situations. In soccer, small-sided games (SSGs) are considered one of the better resources for this purpose [11]. SSGs are modified games played on reduced pitch areas using adapted rules and involving smaller numbers of players compared to the official match play [3, 12, 13]. Pitch size, player number, inclusion of goalkeepers, duration of bouts, coach encouragement, number of touches allowed and the method of defending are some of the studied variables in SSGs [3, 14–16]. Despite the fact that SSGs have been widely studied and reported in the specialist literature [3, 13, 16–19], most of the studies have been carried out in a possession format where the players had to maintain ball possession or obtain one goal with a specific position according to the game system. Taking into account that SSGs are among the exercises most frequently used by soccer coaches [11, 12, 20], they have received many modifications. One of the most novel modifications is the creation of position games. These drills are SSGs where the soccer players have to perform according to a specific role position, trying to reproduce similar situations as in the competition. Although they are daily used in the practice of professional soccer teams, their running requirements are unknown. For this reason, the aims of this study were (1) to compare the running performance of possession and position games and (2) to describe the external load of the same position game played in different dimensions.

## MATERIALS AND METHODS

### Experimental approach to the problem

A descriptive design was adopted to compare running demands of two kinds of sided games. Possession and position games were developed through different formats to maintain ball possession. In possession games the players could move without a standardized role position while in the position games they had to follow their specific role position assigned in the official games. Taking into account that these drills were frequently used during the sessions of the teams, it was not necessary to perform familiarization sessions. The training load of both sided games was monitored to characterize these drills.

### Subjects

Twenty-five young soccer players of the second team of a professional Spanish first division team participated in this study (age: 21.9 ± 1.9 years; height: 177.9 ± 5.2 cm; weight: 75.5 ± 4.8 kg; % body fat (Faulkner): 11.1 ± 1.4%). A total of 656 individual data of outfield players (goalkeepers excluded) were included in the analysis. Players were categorised into five individual playing positions: central defenders (CD), fullbacks (FB), defensive midfielders (DM), offensive midfielders (OM), wide midfielders (WM) and strikers (S). All players were informed about the purpose of the study and each player provided written informed consent. All procedures were approved by the ethical committee of the local university according to the principles of the Declaration of Helsinki for studies involving human subjects.

### Procedures

A descriptive design of 36 training sessions to compare the external load of two types of sided games (possession and position game) was adopted during the 2021–2022 season. Soccer players were divided by the coach into two teams of 9 according to their playing position and a subjective skill assessment of them to allocate players to balanced SSG teams. Moreover, two more players occupied a floater role, always playing with the team in possession of the ball. All sessions started with a 20-min standardized warm-up based on running, ball possession, and dynamic stretching exercises, after which the studied drills were developed. The tasks were performed using a continuous format of 8 min and played with a maximum of two touches per player. These games were presented in a randomized sequence on different days of the week over a period of 36 weeks, according to the tactical and physical requirements of the microcycle. Coaches were required to give verbal encouragement and constructive feedback and to introduce balls immediately when the ball left the playing field.

### Possession games and position games

**FIG. 1:**
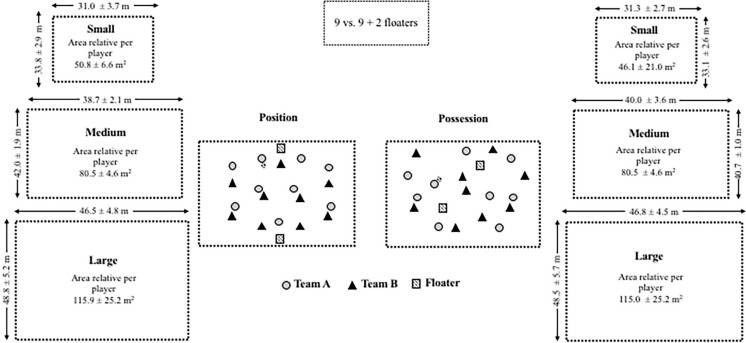
Graphical representation of possession and position games.

The small-sided games implemented in this study are represented in Figure 1. Players were grouped in two teams of 9 with 2 offensive floater players and they performed two formats: possession and position game. The main difference between the formats was that during the possession games (n = 18) the players were moved without a standardized role position, while in the position games (n = 18) they had to follow their specific role position assigned in a 1-4-2-3 system of play (a variant of the 1-4-3-3 development with 9 players in the team and 1 goalkeeper) and according to the previous explained role position. Independently of format, all tasks were developed to maintain the ball possession. Each format of these games was developed in three sizes, classified according to their relative area per player (ApP) as: small (< 60 m^2^ per payer), medium (60–90 m^2^ per player) and large (> 90 m^2^ per player) with six measures made for each size and format (see Figure 1).

### Running activity

Running activity was monitored using a GPS system (WIMU Pro, RealTrack Systems, Almería, Spain) with a sampling rate of 10 Hz. The validity and reliability of this device have been analysed for the collection of time-motion variables, and it is considered a suitable instrument for this purpose in football [21, 22]. Total distance covered (DC), distance covered above 21 km · h^−1^ (DC > 21 km · h^−1^), peak speed, maximal accelerations and decelerations, number of accelerations (Acc_COUNT_) and decelerations (Dec_COUNT_), accelerations lower and higher than 3 m · s^−2^ (Acc_<3_; Acc_>3_, respectively) and decelerations lower and higher than -3 m · s^−2^ (Dec_<3_; Dec_>3_, respectively), and player load were recorded. These variables have been used in the previous literature [19, 23–25].

### Statistical analyses

A descriptive design was adopted to compare running demands of two kinds of small-sided games. To confirm the data normality of each dataset, the Kolmogorov-Smirnov test, the Q-Q plot of residuals and the random coefficients histogram were used. Data not following a normal distribution were transformed before further analysis [26]. General linear model analyses were used to compare the effects of space size (i.e., large, medium, small) of the position and possession tasks on the dependent parameters. The model used for each dependent parameter had size and type of task as independents fixed factors. The ANOVA omnibus test was used to assess the goodness of fit of the models. When a significant main effect was obtained, Bonferroni’s correction was used for multiple comparison analysis. To assess between-task differences for each space size, simple effects were calculated with type of task as the simple effects variable and space size as the moderator variable. Statistical significance was set at α < 0.05. Unless otherwise stated, all values are presented as the estimated marginal mean (SE) or estimated marginal mean and 95% confidence interval (CI). The data analysis was performed using JAMOVI for Mac [27] (version 2.3.16) and the jamovi module GAMLj: General Analyses for the Linear Model in Jamovi [28].

## RESULTS

**FIG. 2:**
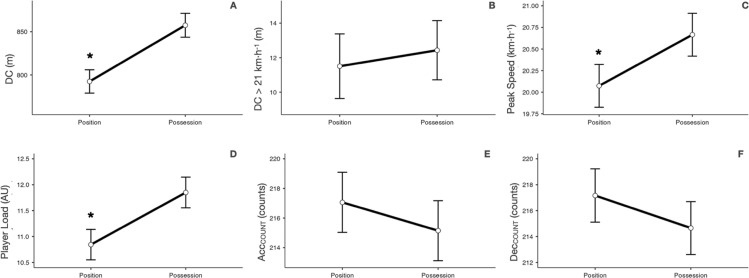
Comparison of possession and position games in A) DC, B) DC > 21 km · h^−1^, C) peak speed, D) player load, E) Acc_COUNT_ and F) Dec_COUNT_. DC = distance covered; DC > 21 km · h^−1^ = distance covered above 21 km · h^−1^; peak speed = highest speed reached; player load = vector magnitude representing the sum of accelerations recorded in the anterior-posterior, mediolateral and vertical planes of movement; Acc_COUNT_ = number of accelerations; Dec_COUNT_ = number of decelerations; * = p ≤ 0.05 statistically significantly different from possession task.

**FIG. 3:**
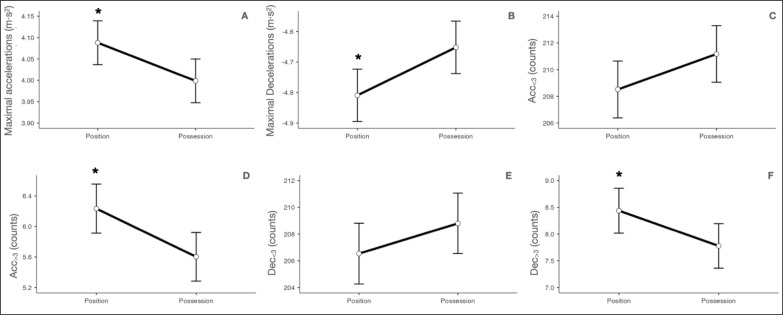
Comparison of possession and position games in A) maximal accelerations, B) maximal decelerations, C) Acc_<3_, D) Acc_>3_, E) Dec_<3_ and F) Dec_>3_. Maximal accelerations = maximal accelerations reached; maximal decelerations = maximal decelerations reached; Acc_<3_ = accelerations lower than 3 m · s^−2^; Acc_>3_ = accelerations higher than 3 m · s^−2^; Dec_<3_ = decelerations lower than -3 m · s^−2^; Dec_>3_ = decelerations higher than -3 m · s^−2^. * = p ≤ 0.05 statistically significantly different from possession task.

Descriptive statistics of the possession and position games are presented in Table 1 and Figures 2 and 3. When comparing the type of task, position games obtained significantly lower values than possession games in DC (mean difference [MD] = -65.04 ± 9.83 m, *p* < 0.001), peak speed (MD = -0.59 ± 0.18 km · h^−1^, *p* < 0.001) and player load (MD = -1.01 ± 0.21 AU, *p* < 0.001) variables. However, position games obtained significantly higher values than Possession games in maximal acceleration (MD = 0.09 ± 0.04 m · s^−2^, *p* = 0.016), maximal deceleration (MD = 0.16 ± 0.06 m · s^−2^, *p* = 0.011), Acc_>3_ (MD = 0.63 ± 0.23 counts, *p* = 0.006) and Dec_>3_ (MD = 0.66 ± 0.30 counts, *p* = 0.029) variables. See Table 1 and Figures 2 and 3.

**TABLE 1:** Running demands of possession and position games.

Variable	Possession	Position
DC (m)	857.53 ± 7.01	792.49 ± 6.89^*^
DC > 21 km · h^−1^ (m)	12.43 ± 0.87	11.51 ± 0.95
Peak Speed (Km · h^−1^)	20.67 ± 0.13	20.07 ± 0.13^*^
Player Load (AU)	11.85 ± 0.15	10.84 ± 0.15^*^
Acc_COUNT_(counts)	215.15 ± 1.03	217.06 ± 1.03
Dec_COUNT_(counts)	214.66 ± 1.04	217.17 ± 1.05
Maximal accelerations (m · s^2^)	4.00 ± 0.03	4.09 ± 0.03^*^
Maximal decelerations (m · s^2^)	-4.65 ± 0.04	-4.81 ± 0.04^*^
Acc_<3_(counts)	211.18 ± 1.08	208.52 ± 1.09
Acc_>3_(counts)	5.60 ± 0.16	6.23 ± 0.16^*^
Dec_<3_(counts)	208.80 ± 1.15	206.54 ± 1.16
Dec_>3_(counts)	7.78 ± 0.21	8.44 ± 0.21^*^

DC = distance covered; DC > 21 km · h^−1^ = distance covered above 21 km · h^−1^; Peak Speed = highest speed reached; Player load = vector magnitude representing the sum of accelerations recorded in the anterior-posterior, mediolateral and vertical planes of movement; Acc_COUNT_ = amount of accelerations; Dec_COUNT_ = amount of decelerations; Maximal accelerations = Maximal accelerations reached; Maximal decelerations = Maximal decelerations reached; Acc_<3_ = Accelerations lower than 3 m · s^−2^; Acc_>3_ = Accelerations higher than 3 m · s^−2^; Dec_<3_ = Decelerations lower than -3 m · s^−2^; Dec_>3_ = Decelerations higher than -3 m · s^−2^.

* = p ≤ 0.05 statistically significant different from possession task.

**FIG. 4:**
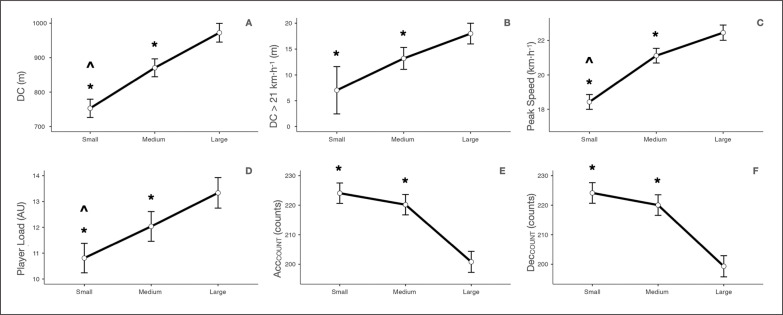
Comparison between sizes of position games in A) DC, B) DC > 21 km · h^−1^, C) peak speed, D) player load, E) Acc_COUNT_ and F) Dec_COUNT_. DC = distance covered; DC > 21 km · h^−1^ = distance covered above 21 km · h^−1^; peak speed = highest speed reached; player load = vector magnitude representing the sum of accelerations recorded in the anterior-posterior, mediolateral and vertical planes of movement; Acc_COUNT_ = number of accelerations; Dec_COUNT_ = number of decelerations; small: small size of the task’s space; medium: medium size of the task’s space; large: large size of the task’s space. *: *p ≤* 0.05 statistically significantly different from large size of the task’s space; ^: *p ≤* 0.05 statistically significantly different from medium size of the task’s space.

**FIG. 5:**
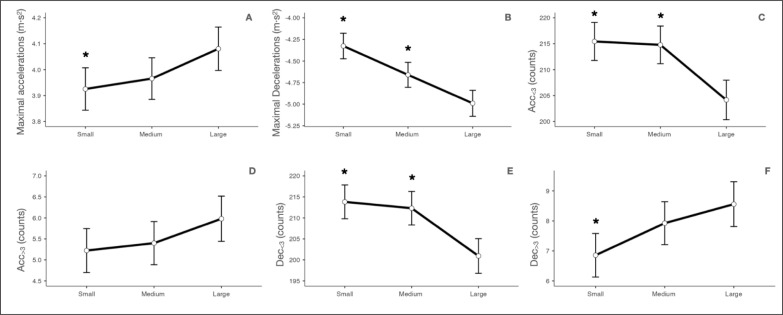
Comparison between sizes of position games in in A) maximal accelerations, B) Maximal decelerations, C) Acc_<3_, D) Acc_>3_, E) Dec_<3_ and F) Dec_>3_. Maximal accelerations = maximal accelerations reached; maximal decelerations = maximal decelerations reached; Acc_<3_ = accelerations lower than 3 m · s^−2^; Acc_>3_ = accelerations higher than 3 m · s^−2^; Dec_<3_ = decelerations lower than -3 m · s^−2^; Dec_>3_ = decelerations higher than -3 m · s^−2^. Small: small size of the task’s space; medium: medium size of the task’s space; large: large size of the task’s space. *: *p ≤* 0.05 statistically significantly different from large size of the task’s space; ^: *p ≤* 0.05 statistically significantly different from medium size of the task’s space.

Running demands of the position games at the three different sizes studied are shown in Table 2 and Figures 4 and 5. When comparing the size of the task space, a significant main effect was found in all variables (p ≤ 0.05) except in Acc_>3_ (p = 0.118). See Table 2 and Figures 4 and 5.

**TABLE 2:** Running demands of the different sizes from position games.

Variable	Small	Medium	Large
DC (m)	752.92 ± 13.51^*^^^^	870.68 ± 13.20^*^	972.31 ± 3.76
DC > 21 km · h^−1^ (m)	7.03 ± 2.32^*^	13.20 ± 1.07^*^	17.99 ± 1.01
Peak Speed (Km · h^−1^)	18.43 ± 0.22^*^^^^	21.12 ± 0.22^*^	22.45 ± 0.22
Player Load (AU)	10.81 ± 0.29^*^^^^	12.04 ± 0.29^*^	13.34 ± 0.30
Acc_COUNT_ (counts)	224.08 ± 1.75^*^	220.18 ± 1.75^*^	200.80 ± 1.81
Dec_COUNT_ (counts)	224.16 ± 1.77^*^	220.05 ± 1.77^*^	199.30 ± 1.82
Maximal accelerations (m · s^2^)	3.93 ± 0.04^*^	3.97 ± 0.04	4.08 ± 0.04
Maximal decelerations (m · s^2^)	-4.33 ± 0.08^*^^^^	-4.66 ± 0.07^*^	-4.99 ± 0.08
Acc_<3_ (counts)	215.44 ± 1.86^*^	214.79 ± 1.84^*^	204.15 ± 1.94
Acc_>3_ (counts)	5.22 ± 0.27	5.40 ± 0.26	5.98 ± 0.27
Dec_<3_ (counts)	213.82 ± 2.04^*^	212.31 ± 2.03^*^	200.93 ± 2.10
Dec_>3_ (counts)	6.85 ± 0.37^*^	7.93 ± 0.36	8.56 ± 0.38

DC = distance covered; DC > 21 km · h^−1^ = distance covered above 21 km · h^−1^; Peak Speed = highest speed reached; Player load = vector magnitude representing the sum of accelerations recorded in the anterior-posterior, mediolateral and vertical planes of movement; Acc_COUNT_ = amount of accelerations; Dec_COUNT_ = amount of decelerations; Maximal accelerations = Maximal accelerations reached; Maximal decelerations = Maximal decelerations reached; Acc_<3_ = Accelerations lower than 3 m · s^−2^; Acc_>3_ = Accelerations higher than 3 m · s^−2^; Dec_<3_ = Decelerations lower than -3 m · s^−2^; Dec_>3_ = Decelerations higher than -3 m · s^−2^. Small: small size of the task’s space; Medium: medium size of the task’s space; Large: large size of the task’s space.

* *p* ≤ 0.05 statistically significant different from large size of the task’s space;

^ *p* ≤ 0.05 statistically significant different from medium size of the task’s space.

Small size was associated with significantly lower values than medium size in DC (MD = -117.77 ± 18.89 m, *p* < 0.001), peak speed (MD = -2.69 ± 0.31 km · h^-1^, *p* < 0.001) and player load (MD = -1.23 ± 0.41 AU, *p* = 0.010) variables. However, small size was associated with significantly higher values than medium size in the maximal decelerations variable (MD = 0.33 ± 0.11 m ·s^-2^, *p* = 0.005)

Small size was associated with significantly lower values than large size in DC (MD = -219.40 ± 19.28 m, *p* < 0.001), DC > 21 km · h^−1^ (MD = -10.96 ± 2.53 m, *p* < 0.001), peak speed (MD = -4.02 ± 0.31 km · h^-1^, *p* < 0.001), maximal accelerations (MD = -0.16 ± 0.06 m ·s^-2^, *p* = 0.028), Dec>_3_ (MD = -1.70 ± 0.53 counts, *p* = 0.004) and player load (MD = -2.53 ± 0.42 AU, *p* < 0.001) variables. However, small size was associated with significantly higher values than large size in ACC_COUNT_ (MD = 23.28 ± 2.52 counts, *p* < 0.001), DEC_COUNT_ (MD = 24.86 ± 2.53 counts, *p* < 0.001), maximal decelerations (MD = 0.66± 0.11 m ·s^-2^, *p* < 0.001), Acc<_3_ (MD = 11.29 ± 2.69 counts, *p* < 0.001) and Dec_<3_ (MD = 12.89 ± 2.93 counts, *p* < 0.001) variables.

Medium size was associated with significantly lower values than large size in DC (MD = -101.63 ± 19.07 m, *p* < 0.001), DC 21 km · h^−1^ (MD = -4.79 ± 1.47 m, *p* = 0.004), peak speed (MD = -1.33 ± 0.31 km · h^-1^, *p* < 0.001) and player load (MD = -1.30 ± 0.42 AU, *p* = 0.006) variables. However, medium size was associated with significantly higher values than large size in Acc_COUNT_ (MD = 19.39 ± 2.52 counts, *p* < 0.001), Dec_COUNT_ (MD = 20.75 ± 2.53 counts, *p* < 0.001), maximal decelerations (MD = -0.33 ± 0.11 m ·s^-2^, *p* = 0.006), Acc_<3_ (MD = 10.64 ± 2.68 counts, *p* < 0.001) and Dec_<3_ (MD = 11.37 ± 2.92 counts/min, *p* < 0.001) variables.

## DISCUSSION

The aims of this study were to compare the running performance of possession and position games and to describe the external load of the same position game played in different dimensions. The main findings were that possession and position games had different running demands showing greater acceleration and deceleration requirements in the possession games and higher DC, peak speed and player load during the position games. Larger size of the position games was associated with higher DC, DC > 21 km · h^−1^, player load and more intense accelerations and decelerations while a smaller size resulted in greater demands of accelerations and decelerations of lower intensity.

The drills monitored in this study showed different features of external loads depending on the inclusion of tactical requirements during SSGs. The presence of a tactical role during the position games forced the soccer players to return to their original position continuously after each movement, which could have led to the higher acceleration and deceleration achieved during these tasks. Likewise, the non-structured format of the possession games where players moved freely without tactical information could have provoked their higher DC, peak speed and player load. During these games there are no any rules restricting the players’ space exploration, which could explain the obtained results. These findings are in line with previous research suggesting that a specific tactical approach may present different performance of the soccer players during matches and training sessions [29–31]. Previously, it was found that different team formations and associated tactical demands had a significant influence on the external load parameters [29], which would explain the dissimilar response of possession and position games. The inclusion of tactical aspects through position games has been shown to be an effective alternative to modify the external load of the traditional SSGs without changing other constrains. Our results reinforce the idea that tactical instructions regarding team formation is one of the most efficient instruments for coaches to change the players’ running behaviour [32]. Furthermore, taking into account that the current knowledge regarding the load experienced by professional soccer players performing SSG-tactical-conditioned training approaches is still scarce [31], these findings could shed some light on this topic, helping coaches in planning training.

In the literature it has been reported that one of the main variables influencing training load during small-sided games is the play area [12, 33]. Different configurations through the pitch size and ApP [34–36] are considered the most important element to manipulate the load of the soccer players in these drills. Although these aspects have been extensively investigated in SSGs [37, 38], to the authors’ knowledge the influence of pitch modification in the external load of position games has not yet been studied.

This study found that when the pitch size of the position games was increased, the soccer players experienced greater DC, running demands at higher speed, more intense change of velocity and higher player load; on the other hand, a smaller pitch size was associated with more accelerations and decelerations of lower intensity. These outcomes are in line with the previous literature published on SSGs [12, 13, 33, 39]. Smaller pitches lead to shorter distances between teammates and opponents [40], which could elicit more non-maximal accelerations and decelerations due to the smaller amount of space developed during the small and medium position games. An interesting study [41] conducted with youth players also found that on a small pitch, more transitions were produced, while on a large pitch, longer ball possessions were more frequent. This would stimulate the presence of this mechanical load during the smaller positional games. Similarly, a recent study confirmed that interventions with ApP < 85.17 m^2^ are likely not large enough to accumulate running and acceleration/deceleration efforts of high intensity [39]. This aspect could explain the decreased load observed during small and medium position games, where the ApP was around 50 and 80.5 m^2^, respectively, compared to 115 m^2^ during the large position games. Previous researchers have suggested that on larger pitches, there may be more distance covered in high-speed running and more intense actions [15, 39, 42]. The increased available space in larger position games could potentially allow soccer players to accumulate a higher workload. Taking into account that the ApP influenced the running demands of the position games, soccer coaches should understand that this knowledge could help them to control and plan their training sessions. Besides its contribution to the training process in soccer, the current study has limitations that must be considered. Firstly, a unique format of a positional game with nine players on each team was employed. However, the study did not indicate the number of players designated as starters, substitutes, or bench players within each group. Additionally, two floaters participated in the positional games as goalkeepers, and considering the offside rule, one of them could have received the ball while in an offside position. With these considerations in mind, future studies should explore various formats of positional games, such as reducing the number of players on each team or adopting a competition format of 10 vs. 10. These studies should specify the players’ status, exclude floaters, or consider variations in the offside rule.

## CONCLUSIONS

In conclusion, practitioners should be mindful that the use of possession and position games, as well as their size, can modify the external load on soccer players during training sessions. Possession and position games exhibit different running requirements, with position games showing greater demands for accelerations and decelerations, and possession games displaying higher DC, peak speed, and player load. Variations in the size of position games are associated with a specialization of the load, resulting in higher DC, DC > 21 km · h^−1^, player load, and more intense accelerations and decelerations in larger formats, while smaller position games involve greater accelerations and decelerations of lower intensity.
